# Case of Ununited Fracture, Cured by Subcutaneous Perforation

**Published:** 1853-05

**Authors:** Harvey Jewett

**Affiliations:** Canandaigua, N. Y.


ART. V.— *Case of Ununited Fracture, cured by Subcutaneous Perforation.* By HARVEY JEWETT, M. D., Canandaigua, N. Y.

The discrepancy of opinion that exists among surgeons on the subject of treating ununited fracture of bones, and the failures that have attended the various plans proposed for their consolidation, have induced me to add the testimony of another case of subcutaneous perforation of the ends of the bone, proposed by Prof. Brainard, of Rush Med. College.

Dr. B. reports a case in the May No. for 1852, of the Buffalo Med. Jour., successfully treated in this way after a failure with the seton, which is the only case we have on record treated in this manner.

Prof. Sandford, of Davenport, Iowa, gives two cases of ununited fracture, which were cured by subcutaneous scarification of the ends of the fragments, which more nearly resembles the mode proposed by Prof. Brainard, than any other recorded case that has come under my observation.

The simplicity, safety, and ease, with which this operation is performed, compared with other modes proposed for the relief of such conditions, if it be equally effectual, certainly commends itself to our consideration and regard.

The pain of the operation is comparatively slight; it is attended with no hæmorrhage, and the suppurative process, which tends to convert the effused blastema into pus, is wholly obviated.

The result of this simple experiment thus far, has been highly satisfactory, and I trust will be faithfully tested whenever such cases present themselves to the surgeon.

Mr. P., aged 30, on the 31st Sept., had both bones of the leg broken by being caught under a large stone; the tibia, one-third distant from the upper, and the fibula, one-third from the lower extremity. The ordinary dressings were applied at the time of the accident, and the limb put upon the inclined plane. About the twelfth day after the accident the dressings were loosened by the patient, and were allowed to remain so for three days, when they were again secured by the attending physician.

At the end of the twelfth week the dressings were removed. The ankle and foot were œdematous, the fibula was firm, but not the slightest evidence of bony union in the tibial fracture. I procured a long straight awl, (the ordinary pegging awl being too short for the purpose,) and introduced it between the fragments, and made six or seven perforations in the lower, and without removing it from the skin, bored the end of the upper fragment in the same way. The limb was secured, the patient put upon a generous diet, with an occasional glass of porter. At the end of two weeks, a second perforation was made in the same manner, and the leg firmly secured with splints as before. After ten weeks the dressings were removed and the union was complete.

CANANDAIGUA, March, 1853.

